# Optimized production, Pb(II) adsorption and characterization of alkali modified hydrochar from sugarcane bagasse

**DOI:** 10.1038/s41598-021-01825-y

**Published:** 2021-11-16

**Authors:** Mohamad Ebrahim Malool, Mostafa Keshavarz Moraveji, Jalal Shayegan

**Affiliations:** 1 Department of Chemical Engineering, Amirkabir University of Technology (Tehran Polytechnic), 424 Hafez Avenue, 1591634311 Tehran, Iran; 2 Chemical and Petroleum Engineering Department of Sharif University of Technology, Tehran, Iran

**Keywords:** Pollution remediation, Environmental economics, Chemical engineering, Pollution remediation

## Abstract

Today, sugarcane bagasse (SB) is used for bioethanol and biodiesel production, energy generation, and adsorbent synthesis. The goal of this project is to determine the optimized conditions for producing adsorbent from sugarcane bagasse using hydrothermal carbonization (HTC) and KOH activation. To optimize process parameters such as reaction temperature, residence time, ZnCl_2_/SB mixing ratios, and water/SB mixing ratios, response surface methodology was used. The results revealed that the optimum modified adsorption occurred at 180 °C, 11.5 h, a water to biomass ratio of (5:1), and a ZnCl_2_ to precursor ratio of (3.5:1). The physicochemical features of optimum activated hydrochar were investigated, as well as batch adsorption experiments. The pseudo-second-order kinetic model and the Langmuir isotherm model were found to fit the experimental results in batch adsorption studies [${q}_{max}=90.1$ (mg/g)]. Thermodynamic experiments further confirmed the spontaneous and exothermic adsorption mechanism.

## Introduction

Agricultural biomass waste is made up of organic substances created by humans in agricultural activities. This waste can be used as a raw material for the manufacturing of valuable products such as fuels, biogas, and adsorbents due to its abundance and availability^1,2^. Sugarcane bagasse (SB) is a cellulose, hemicellulose, and lignin-rich agricultural biomass waste. In the past, SB was used for energy generation, but it is now a great substrate for environmentally friendly approaches like bioethanol, energy generation, biodiesel, and adsorbent synthesis^3^. SB can be a suitable candidate for adsorbent production and removing pollutants such as lead from the environment.

Lead is one of the most common and dangerous metals found in smelting, battery, and coating industry effluents. Pb^2+^ can enter the body through the skin, digestive tract, and respiratory tract, causing damage to people (brain, mental, renal, and liver illness, anemia, and other disorders), and will accumulate in mammal bodies, according to research. Chemical precipitation, ion exchange, liquid membrane, electrochemical, and adsorption procedures are among the various ways utilized to remediate lead-polluted wastewater today. Adsorption is a good alternative for heavy metal handling because of its basic equipment, ease of operation, low cost, and acceptable efficiency^4^. Today hydrothermal carbonization is used as a green technology for biomass waste management to create valuable materials such as adsorbents.

Hydrothermal carbonization (HTC) is a primarily exothermic thermochemical conversion process that converts waste biomass into useful materials at a low temperature (180–250 °C) and under autogenous pressure. HTC's products include hydrochar (HC), a carbonaceous coal-like solid material with rich oxygen functional groups, process water (a mixture of bio-oil and water), and a small amount of gases^5,6^. The type of waste biomass used, as well as process parameters such as temperature, resident time, water to biomass ratio, catalyst quantity, and activation method, all influence the characteristics of the HC generated^7^. Water works as a solvent medium, reactant, and catalyst in the HTC process because to the changing physicochemical properties of water at elevated temperatures (ionization strength and dielectric constant). It contributes to waste biomass hydrolysis and cleavage^7,8^.

The raw material undergoes numerous important reactions during the HTC process. The initial step in each reaction is hydrolysis. After that, a series of reactions will be carried out, including dehydration, decarboxylation, condensation, and polymerization^9,10^. HC can be used as a fuel, soil amelioration, and a desirable material for adsorbent material. Because HC has a lower oxygen to hydrogen and hydrogen to carbon ratio (O/H and H/C) and higher oxygenated functional groups (OFGs) than raw material, it can be used as a suitable candidate for production of activated carbons and adsorbents^11,12^.

Activated carbons play an important role in wastewater treatment due to their ease of handling, low processing costs, and higher adsorption effectiveness than other pollutant removal methods^13,14^. HTC of lignocellulosic biomass for the removal of organic and inorganic pollutants from wastewater is a promising technology due to the low cost of feedstock materials, the use of renewable and diverse sources of material, and the ecologically beneficial process.

In recent years, the generation of activated carbon from HTC of biomass such as Cotton stalk^15^, avocado seed^16^, flax shives, and oat hulls^17^, mango peels^18^, palm leaves^19^, cassava slag^1^, brewer's spent grain^20^, corncob^21^, rice straw^22^, Lepironia articulate^23^, banana fruit bunch^24^, hickory wood and peanut hull^25^, bamboo shoot^26^, Teak Sawdust^27^, bamboo sawdust^28^, Agave americana fibres and mimosa tannin^29^, prolifera-green-tide^30^ have been studied.

To the best of knowledge of the authors, no study has looked into the complete impact of process factors (temperature, resident time, biomass to water ratio, and biomass to catalyst ratio) on the adsorption capacity of waste biomass-derived hydrochar. The effect of process factors on the HTC of sugarcane bagasse with alkali alteration was investigated in this experiment, and optimum adsorption conditions were discovered. After then, the properties of activated hydrochar were investigated. The main purpose of this study is to look into the optimized process parameters for HTC of BG based on adsorption capacity and hydrochar yields for the first time.

The study's precise objectives were to:

- Assess the potential of HTC of SB for lead adsorption.
- Using a central composite design-response surface methodology (CCD-RSM) technique, investigate the effect of process variables (temperature, resident time, water to biomass ratio, and catalyst dose) on changed adsorption capacity.
- Performing batch adsorption study to investigate isotherm, kinetic and thermodynamic study, and characterization of optimum adsorbent.

## Results and discussion

### Design of experiment

#### Experimental design and equilibrium adsorption capacity

Table 1 lists the independent factors and response values for each experiment. The CCD design was arranged into 30 runs with 4 variables and five levels, 16 full factorial design matchings, 8 axial trials, and 6 repeats in the central position. The modified adsorption capacity (MAC) of activated hydrochar (AHC) increases from 6.70 ± 0.42 to 53.56 ± 1.98 (mg/g), indicating that HTC parameters had a significant impact on adsorption capacity and yield.

**Table 1:** Independent variables and corresponding response value.

Run	Temperature (°C)		Water to biomass ratio (g/g)		ZnCl_2_ to biomass ratio (g/g)		Resident time (h)		Adsorption capacity (mg/g)	Yield (g/g)	MAC (mg/g)/(g/g)
	Real value	Coded value	Real value	Coded value	Real value	Coded value	Real value	Coded value			
1	230	+ 1	10	+ 1	1.5	− 1	11.5	+ 1	20.75	0.4381	9.09
2	180	− 1	5	− 1	3.5	+ 1	4.5	− 1	52.00	0.6636	34.45
3	180	− 1	10	+ 1	1.5	− 1	11.5	+ 1	54.11	0.5415	29.30
4	230	+ 1	5	− 1	3.5	+ 1	4.5	− 1	40.07	0.5289	21.19
5	180	− 1	10	+ 1	1.5	− 1	4.5	− 1	22.93	0.6360	14.58
6	180	− 1	10	+ 1	3.5	+ 1	11.5	+ 1	86.70	0.5008	43.42
7	205	0	7.5	0	0.5	− 2	8	0	65.92	0.4397	28.99
8	205	0	2.5	− 2	2.5	0	8	0	83.71	0.5667	47.44
9	205	0	12.5	+ 2	2.5	0	8	0	87.73	0.4410	38.66
10	180	− 1	5	− 1	1.5	− 1	11.5	+ 1	77.29	0.5630	43.51
11	205	0	7.5	0	2.5	0	8	0	80.45	0.4892	39.36
12	205	0	7.5	0	2.5	0	1	− 2	10.62	0.8304	8.82
13	230	+ 1	10	+ 1	1.5	− 1	4.5	− 1	25.36	0.4270	10.82
14	230	+ 1	5	− 1	1.5	− 1	4.5	− 1	41.83	0.4694	19.64
15	205	0	7.5	0	4.5	+ 2	8	0	97.33	0.5208	50.70
16	205	0	7.5	0	2.5	0	15	+ 2	76.88	0.5028	38.65
17	205	0	7.5	0	2.5	0	8	0	94.59	0.4944	46.76
18	180	− 1	10	+ 1	3.5	+ 1	4.5	− 1	28.83	0.6307	18.18
19	255	+ 2	7.5	0	2.5	0	8	0	18.44	0.3638	6.70
20	180	− 1	5	− 1	1.5	− 1	4.5	− 1	42.59	0.6753	28.76
21	205	0	7.5	0	2.5	0	8	0	96.84	0.4910	47.55
22	230	+ 1	10	+ 1	3.5	+ 1	4.5	− 1	27.53	0.4542	12.51
23	155	− 2	7.5	0	2.5	0	8	0	22.29	0.6703	14.94
24	205	0	7.5	0	2.5	0	8	0	97.51	0.5044	49.18
25	230	+ 1	5	− 1	3.5	+ 1	11.5	+ 1	32.22	0.5424	17.48
26	205	0	7.5	0	2.5	0	8	0	95.43	0.4921	46.96
27	180	− 1	5	− 1	3.5	+ 1	11.5	+ 1	92.24	0.5808	53.57
28	230	+ 1	10	+ 1	3.5	+ 1	11.5	+ 1	40.05	0.4767	19.09
29	230	+ 1	5	− 1	1.5	− 1	11.5	+ 1	37.48	0.4847	18.17
30	205	0	7.5	0	2.5	0	8	0	98.83	0.4907	48.50

#### Model fitting and analysis of variance (ANOVA)

The hypothesis testing (p-value) and F-test (Fisher test) were used to evaluate significant factors and model fitting. Table S1 (Supplementary information) shows the results of the analysis of variance (ANOVA) for the independent variables temperature (A), water to biomass ratio (B), ZnCl_2_ to biomass ratio (C), and resident time (D). The linear terms A, B, C, and D, as well as the quadratic terms A_2_, C_2_, D_2_, and the synergy term AD, were noteworthy variables. Equation (1) describes the second-order regression equation adjusted to the response (MAC) in the form of coded values after eliminating non-important variables.

1 $$MAC=46.38-8.81A-4.06B+3.73C+5.55D-12.66{A}^{2}-1.83{C}^{2}-5.86{D}^{2}-4.63AD$$

Temperature and resident time were more significant than other factors according to the fitting equation and F-test (Table S1). The most important variable in changed adsorption capacity was temperature (F-value equal 83.80). The temperature and resident time interaction was the most important of the various interactions between the variables (Table S1).

Table S2 shows the ANOVA for the response model. The response model has a reasonable F-value of 27.04 and a p-value of 0.0001 (less than 0.05), as well as a lack of fit p-value of 0.37 (higher than 0.05)^31^.

**Figure 1:**
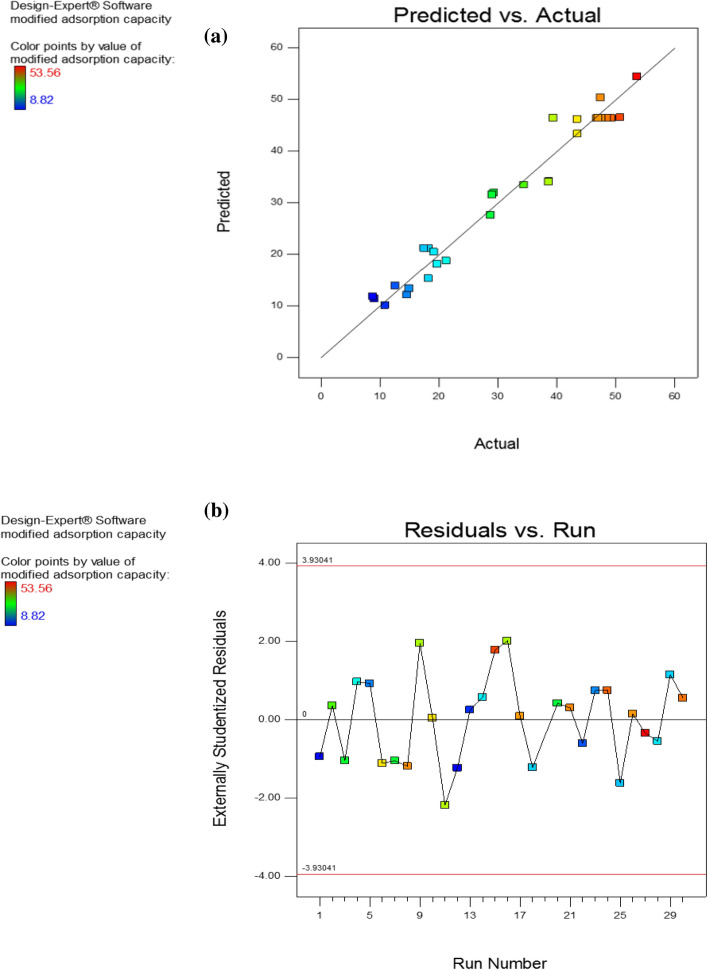
Relationship between the actual (experimental) and predicted values (**a**) and plot of the residues versus experimental order (**b**).

Figure 1 depicted the scattering of residues as well as the link between actual (experimental) and predicted values (Fig. 1a,b). As can be seen in Fig. 1a, the near proximity of the points to the predefined line suggested that the regression model's actual and anticipated values were quite close. As shown in Fig. 1b, with a random distribution and low residue values, the model is substantial^32,33^.

#### Response of surfaces

**Figure 2:**
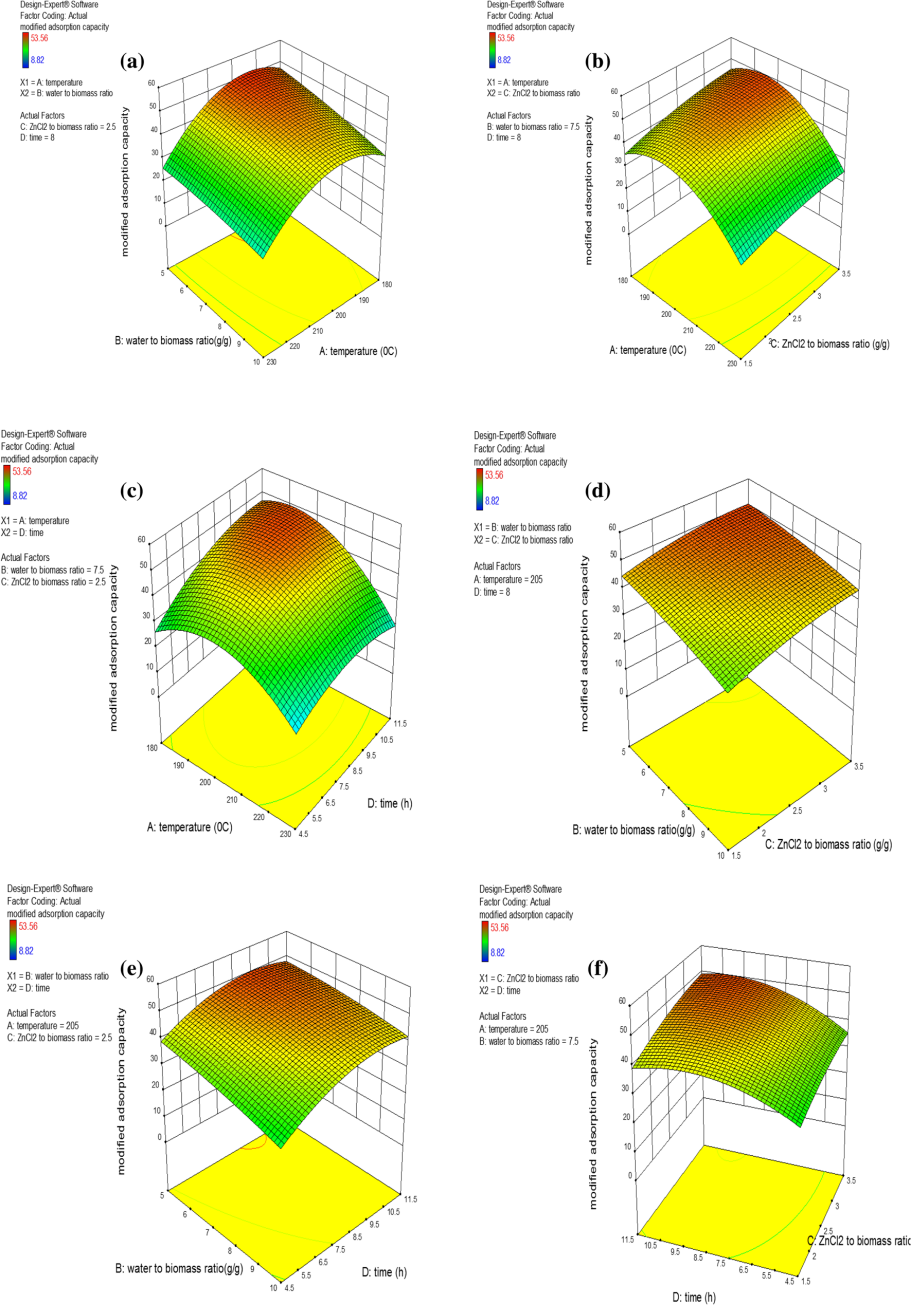
Three-dimensional response surface for MAC: water to biomass ratio versus temperature (**a**), temperature versus ZnCl_2_ to biomass ratio (**b**), temperature to time ratio (**c**), water to biomass ratio versus ZnCl_2_ to biomass ratio (**d**), water to biomass ratio versus time (**e**) and time to ZnCl_2_ to biomass ratio (**f**).

The effects of two-factor interactions (the other variables fixing at coded value of zero) on the MAC were investigated, and the findings are shown in Fig. 2. As shown in Table S1, regarding F-value, temperature is the most important variable, and interactions between temperature and other factors are more relevant than other conceivable interactions. Furthermore, temperature and time played a crucial role in adsorption performance. So the effect of time and temperature (as fixed parameters) on two-factor interactions were represented in Fig. S1. The quantity of oxygenated functional groups (OFGs) in hydrochar based adsorbent influences its adsorption capability. Previous study showed that temperature and resident time influenced the amount of OFGs in HC^7^. The response of the surfaces for synergy terms (AB, AC, …, CD) are shown in Fig. 2. Furthermore, other variables are fixed at zero coded value, and the effect of temperature and resident time on the synergy coefficients is shown in Fig. S1.

The interplay of temperature with the water-to-biomass ratio (other two variable fixed at zero coded value) is depicted in Fig. 2a. The MAC increased and then dropped with rising temperature at any water to biomass ratio. It's possible that the oxygenated functional groups grew as the temperature rose and subsequently reduced at a higher temperature^34,35^. As shown in Fig. S1, the interaction between temperature and water-to-biomass ratio at different times are similar but at a lower resident time, maximum MAC occurred at the higher temperature. The connection between temperature and the ZnCl_2_ to biomass ratio (other two variable fixed at zero coded value) is seen in Fig. 2b. At any ZnCl_2_ to biomass ratio, with rising temperature, the modified adsorption capacity grew, reached its maximum, and then decreased, similar to the previous interaction. As shown in Fig. S1, the interaction between temperature and ZnCl_2_ to biomass ratio are similar at different times, but at a higher resident time, maximum MAC occurred at the lower temperature. The temperature and resident time interaction (other two variable fixed at zero coded value) is represented by Fig. 2c. Similar to the preceding interactions, the maximum adsorption was obtained with increasing temperature at any time, and thereafter it declined^36^. Unlike earlier interactions, time dependency of response decreased as temperature increased, therefore time became more important at lower temperatures. It could be explained as the dependency of OFGs on temperature and time. At a specific temperature, with rising times, the OFGs reached a maximum and then decreased. At higher temperatures, the rate of reactions in the HTC process increased, and maximum OFGs formation and time to reach equilibrium OFGs decreased^7^. The relationship between the water to biomass ratio and the ZnCl_2_ to biomass ratio (other two variable fixed at zero coded value) is shown in Fig. 2d. Compared to other interactions, this one had fewer consequences. The adsorption rose marginally as the amount of ZnCl_2_ to biomass ratio was raised while the amount of water to biomass ratio was decreased. The interaction between the water to biomass ratio and the ZnCl_2_ to biomass ratio at different times and temperatures are shown in Fig. S1. The trend of MAC changing concerning the water to biomass ratio and the ZnCl_2_ to biomass ratio is similar regardless of time and temperature. At higher temperatures, the dependency of MAC on temperature is reduced, but at a lower temperature, with rising time, the MAC is raised. The interplay between time and the water-to-biomass ratio (other two variable fixed at zero coded value) is depicted in Fig. 2e. The adsorption reduced as the water content increased, but at any water to biomass ratio, the reaction reached a maximum and then decreased marginally as time passed. The interaction between the water to biomass ratio and the time at different temperatures is shown in Fig. S1. The trend of MAC changing concerning the water to biomass ratio and time is similar, But with rising temperature, the maximum MAC occurs at a lower resident time. The interplay between time and the ZnCl_2_ to biomass ratio (other two variable fixed at zero coded value) is depicted in Fig. 2f. With increasing time, the adsorption of any ZnCl_2_ ratio increased, then decreased slightly. The interaction between time and the ZnCl2 to biomass ratio at different temperatures is shown in Fig. S1. With rising temperature, the maximum MAC occurs at a lower resident time. At any ZnCl_2_ to biomass ratio, with increasing time, the OFGs formed and reached the maximum. Then they decreased because of transformation OFGs to stable oxygen surface groups by excessive dehydration/carbonization reaction or breakdown of OFGs to the gaseous product^7^.

#### Optimization

Software delivered 100 optimization conditions across a range of experimental research. Economic consideration depicted that the lower temperature is preferable to the higher temperature among the various optimization conditions that maximize responses (MAC).

The optimum temperature is 180 °C, with a water-to-biomass ratio of 5 (w/w), a ZnCl_2_-to-biomass ratio of 3.5 (w/w), and a resident time of 11.5 h. The actual and response values (by employing a quadratic regression model) for (MAC) were 53.56 ± 1.98 and 54.45 (mg/g) at this condition, respectively (relative error equals 1.7 percent). AHCop was the name given to the activated hydrochar created under ideal conditions, and its characteristics were investigated.

### Characterization of adsorbents

#### Physicochemical properties

Table 2 shows the proximate analysis of BG and HC in optimum conditions (HCop). Dehydration and condensation reactions in HTC result in a decrease in the hydrogen and oxygen content of HC rather than biomass as the reactions progress. As a result, the H/C and O/C atomic ratios of HC were found to be lower than those of biomass^7^. Table 2 shows the BET surface area of BG, HCop, and AHCop. Other experiments have confirmed that HC had a limited surface area^37,38^.

**Table 2:** Proximate analysis of bagasse and hydrochar in optimum conditions.

Sample	Elemental composition (%, mass based)					Atomic ratio		S_BET_ m^2^/g
	C	H	N	O^a^	Ash	(O/C)	(H/C)	
BG	43.10 ± 0.72	5.73 ± 0.14	1.99 ± 0.11	42.19 ± 0.29	6.99 ± 0.17	0.98 ± 0.02	0.13 ± 0.01	0.36 ± 0.24
HCop	58.92 ± 0.88	4.62 ± 0.21	2.10 ± 0.07	25.65 ± 0.33	8.71 ± 0.14	0.43 ± 0.01	0.08 ± 0.01	2.22 ± 0.37
AHCop	–	–	–	–	–	–	–	5.99 ± 0.28
BG	Holocellulose				Hemicellulose		Lignin	
	62.55 ± 2.87				27.60 ± 1.12		38.65 ± 1.45	

^a^The content of O is calculated according to the equation: (100 − (C + H + N + ash)).

#### SEM and EDX analysis

**Figure 3:**
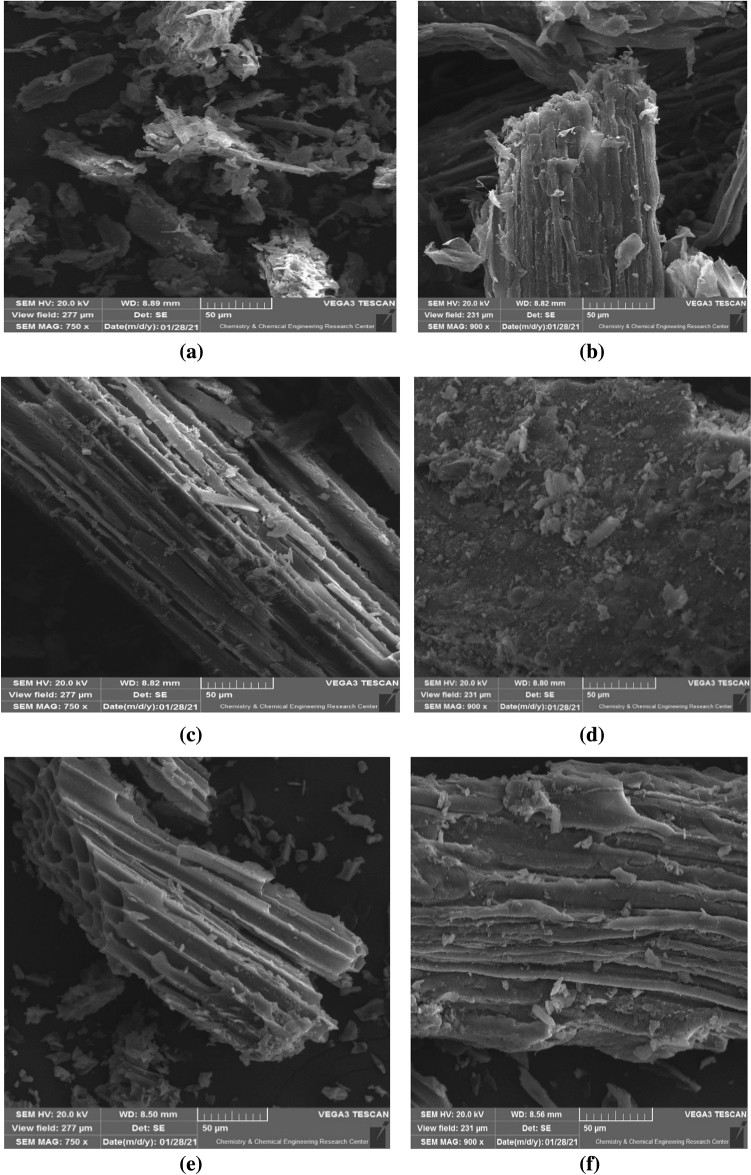
SEM images of (**a**, **b**) bagasse, (**c**, **d**), HCop, and AHCop (**e**, **f**).

Figure 3a–f show the SEM images of BG, HCop, and AHCop, respectively.

The structure of the HCop and AHCop is fairly comparable to the BG due to the low temperature (180 °C) in HTC. HTC generated spongy structures with disordered structural cracks and canals in both HCop and AHCop, it should be noted. Figure 3 revealed that HCop's surface is rougher than BG's, and that AHCop's surface is rougher than HCop's. It's possible that the activation with KOH resulted in the removal of contaminants from partially closed pores, which influenced the growth of surface fractures^39^. The removal of contaminants from pores and the expansion of surface cracks aided adsorbent diffusion and increased adsorption capacity.

Fig. S2 shows an EDX diagram of samples. As can be seen in Fig. S2 (a, b) when bagasse was activated with KOH, the O and C peaks rose and Si peak is diminished. The presence of Pb^2+^ peaks in Fig. S2d,f confirmed Pb(II) adsorption. Because the Pb(II) peaks of AHCop are higher than those of HCop, alkali activation increases adsorption capacity. Regarding Fig. S2, after Pb(II) adsorption, K peaks disappear, followed by the emergence of Pb(II) peaks. It implies that Pb(II) ions replaced K ions.

A significant reduction in Si peaks in HCop and C peak in AHCop, followed by the emergence of Pb(II) peaks, verified the ion-exchange mechanism.

#### XRD analysis

In HTC, XRD is used to investigate crystalline changes in BG. Fig. S3 shows the XRD diagrams of the BG, HCop, and AHCop. Peaks around 16 and 22 degrees confirmed the crystalline structure of BG, as seen in Fig. S3a^40^. The narrow peaks of HCop and AHCop about 21° in Fig. S3b and c indicated incomplete cellulose breakdown during HTC. Peaks in the HCop and AHCop match (0 0 2) graphite structure and demonstrate graphene-like layers at roughly 26°^20^. A diminution of the cellulose peak in AHCop rather than HCop is seen in Fig. S3b and c. KOH activation reduced this peak by causing partial cellulose breakdown^4^. A small peak may also be seen in HCop at about 43°, which corresponds to (1 0 0/1 0 1) and points to the diffraction of graphite layers, however this intensity is destroyed in AHCop due to the KOH activation.

#### FTIR analysis

**Figure 4:**
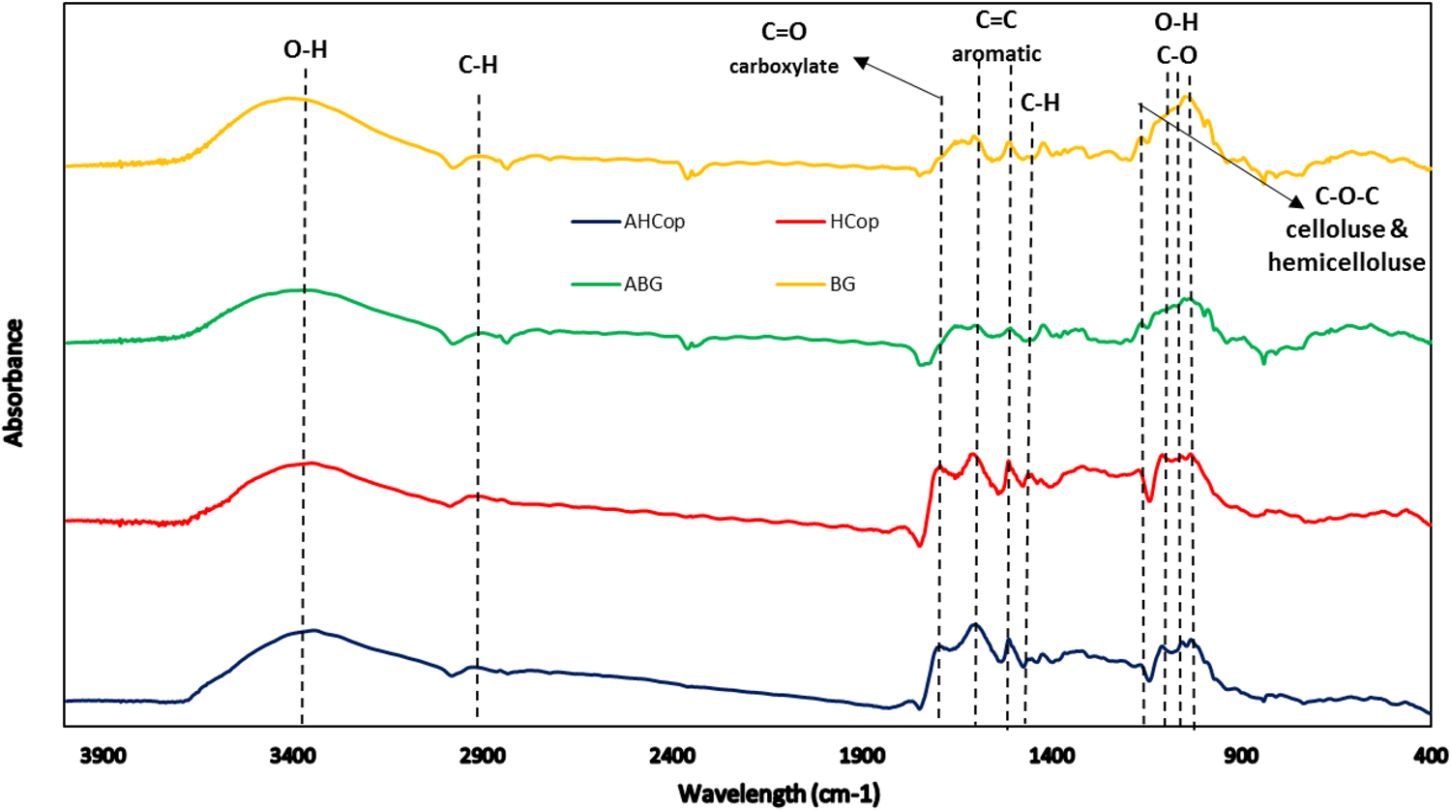
FTIR spectrum of BG, ABG, HCop and AHCop.

The functional groups of materials were studied using the FTIR technique. Figure 4 shows the FTIR spectrum of BG, ABG, HCop, and AHCop. All of the representatives showed the adsorption peaks around 3350, 2920, 1603, and 1513 cm^−1^. The broad-band peaks detected around 3350 cm^−1^ were signed to the O–H stretching vibration of carboxyl or hydroxyl groups. Raw materials that containing a considerable amount of cellulose or hemicellulose could improve the conformation of O–H groups on the surface of HC ^41^. The band peaks located around 2920 cm^−1^ were ascribed to the aliphatic C–H from methyl, methylene, and methine groups ^42^. The peaks discovered at 1603 cm^−1^ and 1513 cm^−1^ were related to C=C stretching vibration of the aromatic structure. The peaks were increased due to dehydration, and aromatization reactions were accomplished in the HTC process^43,44^.

The peaks discovered in HCop and AHCop at 1693 cm^−1^ were related to C=O stretching vibration of carbon groups^45^. The band peaks detected around 1452 cm^-1^ for AHCop and HCop are related to C–H deformation in lignin and carbohydrates^11^. The small peak bands seen in AHCop and HCop in the range of 1300–1000 cm^-1^ were related to O–H and C–O stretching vibrations of carboxyl acid, lactone, ether, and alcohol groups. These bonds did not appear in BG and ABG and will be produced as consequences of the HTC process so that HTC can produce an abundant oxygenated group on the surface of HC^46,47^. It must be mentioned that C–O, C=C, and O–H groups have a considerable effect through adsorption of Pb^2+^ on the surface of adsorbent. The carboxyl (–COOH) and/or hydroxyl (–OH) functional groups can be linked with Pb^2+^ due to the surface complexation. The heterocyclic compounds (C=C) have the electron donation ability. So, these compounds can be able for electrostatic interaction in Pb^2+^ adsorption^48^. The band appeared around 1161 cm^−1^ related to C–O–C cellulose and hemicellulose; after HTC, this group was weekend^41^.

#### TGA analysis

**Figure 5:**
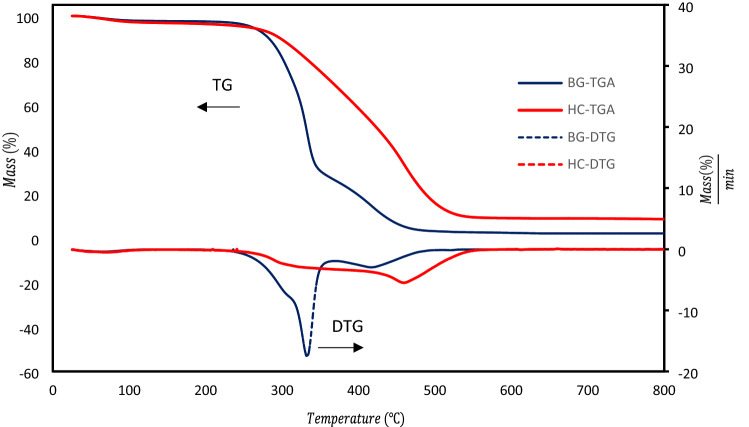
TG and DTG diagram of bagasse and HCop.

Figure 5 shows the TG and DTG curves resulting from the thermal degradation of BG and HCop at a heating rate of (10 °C/min). Table 3 shows their pyrolysis parameters, which include T_v_, T_f_, T_m_, DTG_m_, R_m_, and the temperature range of pyrolysis. During the pyrolysis of BG, three primary decomposition stages were identified, as illustrated in Fig. 5. The evaporation of inherent water has occurred at temperatures lower than 210 °C. The breakdown of hemicellulose was primarily connected with the first stage, which occurred at temperatures ranging from 219.9 to 308.1 °C and resulted in a weight loss of 28.26 percent^49^.

**Table 3:** Pyrolysis characteristic parameters of BG and the AHCop at heating rate of 10 °C/min*.*

Sample	T_v_ (°C)^a^	T_f_ (°C)^b^	T_m_ (°C)^c^	DTG_m_ (%/min)^d^	R_m_ (%)^e^	Stage 1 temperature range (°C)	Stage 2 temperature range (°C)	Stage 3 temperature range (°C)
BG	239.7	509.2	333	17.54	2.12	219.2–308.1	308.1–370.9	370.9–509.2
HCop	236.1	576.8	460.2	5.49	8.52	231.5–387.8	387.8–578	–

^a^T_v_: the initial decomposition temperature.

^b^T_f_: the terminated temperature.

^c^T_m_: the maximum decomposition rate temperature.

^d^DTG_m_ is maximum weight loss rate.

^e^R_m_ is final mass weight.

The second stage, which took place at a temperature of 308–370 °C and resulted in a weight loss of 49.67 percent, saw the majority of the cellulose and some lignin degrade^50^. The third stage took place at a temperature of 370.9–504.5 °C and resulted in a weight loss of 22.78 percent due to lignin breakdown. The TGA and DTG diagrams of HCop compared to BG revealed apparent differences. In contrast to BG, the quantity of weight loss of HCop decreased at temperatures between 250 and 360 °C, while it rose at temperatures between 360 and 500 °C. As a result, during HTC, it may be regarded as a decrease in volatile matter and an increase in fixed carbon^51,52^.

The first decomposition temperature of HCop compare to BG increased from 239.7 to 247.8 °C, as indicated in Table 3; this was perhaps due to a decrease in volatile materials during HTC^53^. HTC also increased the final pyrolysis temperature from 509.2 to 583.7 °C. The DTG_m_ was reduced from 17.54 (percent/min) for BG to 5.49 (percent/min) for HCop. Furthermore, because to increased ash and decreased volatile matter, the HCop exhibited greater T_m_ and R_m_ than the BG^54^.

#### Adsorption study

The equilibrium adsorption capacity of BG, ABG, HCop, and AHCop was shown in Table 4. It is cleared that KOH activation increased the adsorption capacity of HC from 34.59 ± 2.27 to 92.24 ± 3.41 (mg/g) (about three-fold increased). Also, BG adsorption capacity increased about five-fold with HTC, followed by KOH activation.

**Table 4:** Equilibrium adsorption capacity of BG, ABG, HCop, and AHCop at 25 °C.

Samples	Equilibrium adsorption capacity (mg/g)
BG	20.87 ± 1.62
ABG	28.46 ± 1.87
HCop	34.59 ± 2.27
AHCop	92.24 ± 3.41

#### Effect of initial pH

**Figure 6:**
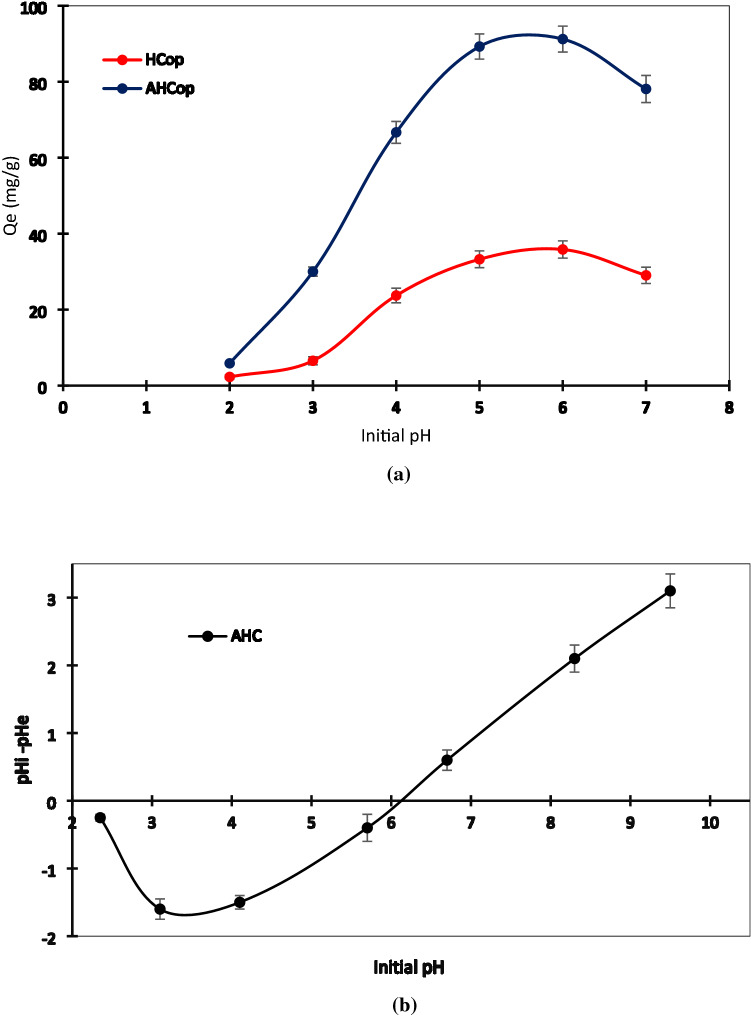
Effect of pH on adsorption of Pb^2+^ onto AHCop and HCop (**a**); Point of zero charge of AHCop (**b**). Error bars represent the standard deviation of three repeated experiments.

Because of the low surface area of adsorbent and poor porosity of HCop and AHCop, the mechanism responsible for Pb^2+^ sorption can be illustrated by surface complexation and electrostatic interactions. At a low pH value, H^+^ is more favorable than lead for electrostatic interaction, and complex formation of OFGs with Pb ions can't be accomplished^48,55^. In Fig. 6a, the effect of initial pH on adsorption is depicted. At different pH levels, AHCop and HCop showed similar adsorption patterns. As shown in Fig. 6a, with increasing pH (reduction of H^+^ ions in solution), the complexation reaction between surface and Pb(II) ions can be accomplished. Moreover, the amount of H^+^ ions was reduced, and Pb(II) ions can be absorbed by the active site that had been occupied with the H + ions at a lower pH value. At a pH of 6 ± 0.1, the AHCop and HCop had maximum adsorption of 92.24 ± 3.41 and 34.59 ± 2.27 (mg/g), respectively. The Pb(II) ions and H^+^ compete for adsorption on the HC surface (electrostatic attraction). H^+^ adsorption is more favorable than Pb(II) adsorption at lower pH value. Also, at higher H^+^ concentrations, the complex formation of OFGs and Pb(II) ions is difficult. Deprotonation of functional groups happens as the pH value rises, Pb(II) ions substitute H^+^, and complex formation occurs, and finally, Pb adsorption increases^56^.

The pH_pzc_ of AHCop was about 6.1 ± 0.1, as indicated in Fig. 6b. Because of the positive surface charge of the sorbent, removing anions is easier at pH values lower than pH_pzc_. The adsorption of cations is more advantageous at higher pH values^1,57^. The repulsive electrostatic forces greatly affect the adsorption of Pb^2+^ onto the AHCop surface since maximal adsorption capacity occurred at a pH value of 5.7 ± 0.1. Moreover, the adsorption capacity is affected by initial pH value^4,57^.

#### Adsorption kinetics study

**Figure 7:**
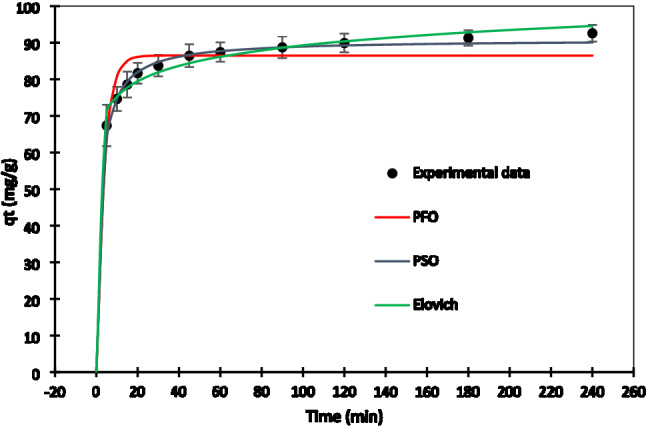
Kinetic data from Pb^2+^ adsorption onto AHCop (T = 25 °C). Error bars represent the standard deviation of three repeated experiments.

Figure 7 depicts the kinetics of Pb^2+^ adsorption by AHCop. In the first 20 min, a significant amount of Pb^2+^ adsorption happened immediately, and then it gradually grew to the equilibrium value. Regarding Fig. 7, the adsorption capacity is varied smoothly after 90 min, maintaining constant around 90 (mg/g), indicating that equilibrium had occurred.

The abundance of adsorption sites on the AHCop was linked to the higher Pb^2+^ adsorption capacity in the first 20 min. The number of free adsorption sites dropped over time, while the adsorption capacity remained stable^58^. AHCop is a suitable adsorbent for Pb^2+^ removal due to its appropriate adsorption kinetics and acceptable adsorption capacity. Table 5 compares Pb^2+^ adsorption capacities among other HC-based adsorbents from other studies and the present study^4,55,59–62^. Table 6 shows the computed kinetic coefficients and adsorption capacities of the PFO, PSO, and Elovich kinetic models. The regression coefficient (R^2^) and Adjusted R-squared (R^2^_Adj_) of the PSO model is substantially higher than that of the PFO and Elovich models. Also, Residual sum of squares (RSS) and reduced chi square (χ^2^_red_) of PSO model is lower than PFO and Elovich models, as shown in Table 6. As a result, the PSO model suited the experimental data better, showing that chemical adsorption was the main process^63^.

**Table 5:** Comparison of Pb^2+^ adsorption capacities among other HC-based adsorbents from other studies and the present study*.*

Adsorbent	Maximum Pb^2+^ adsorption capacities (mg/g)	References
Peanut hulls hydrochar (modified H_2_O_2_)	22.82	^59^
Prospis Africana shell hydrochar	45.3	^55^
grape pomace hydrochar (KOH activated)	137	^4^
Arecanut husk biomasshydrochar	79.86	^60^
Sewage sludge hydrochar/MgAl-layered double hydroxides composites	62.4	^61^
Eupatorium adenophorum hydrochar (HNO_3_ modified)	164.68	^62^
Bagasse based hydrochar (KOH activation)	92.24	Present

**Table 6:** Kinetics parameters for Pb^2+^ adsorption on the optimum activated hydrochar (AHC_op_).

Model	Parameter	Value
PFO ${q}_{t}={q}_{eq}[1-\mathrm{exp}\left(1-{k}_{1}t\right)]$	${q}_{eq}$ (mg/g)	86.851
	${k}_{1} ({\mathrm{min}}^{-1})$	0.254
	R^2^	0.697
	R^2^_adj_	0.621
	RSS	187.248
	${\upchi }_{9}^{2}$	0.253
PSO ${q}_{t}=\frac{{k}_{2}{q}_{eq}^{2}t}{1+{k}_{2}{q}_{eq}}$	${q}_{eq}$ (mg/g)	93.460
	${k}_{2}$	0.003
	${R}^{2}$ (g/mg min)	0.988
	R^2^_adj_	0.985
	RSS	9.424
	${\upchi }_{9}^{2}$	0.014
Elovich ${q}_{t}=\frac{1}{b}\mathrm{ln}(1+abt)$	$b$ (g/mg)	0.016
	$a$ (mg min/g)	1.426 × 10^+5^
	${R}^{2}$	0.942
	R^2^_adj_	0.928
	RSS	33.665
	${\upchi }_{9}^{2}$	0.047

#### Adsorption isotherms study

**Figure 8:**
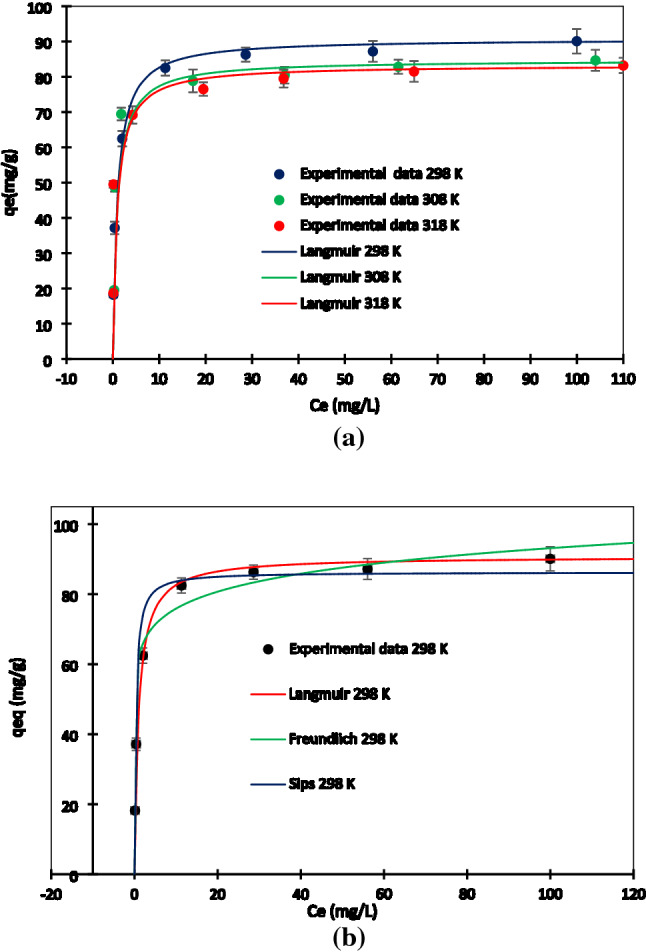
Equilibrium data from Pb^2+^ adsorption onto AHCop. Error bars represent the standard deviation of three repeated experiments.

Pb^2+^ adsorption isotherms are shown on Fig. 8. Figure 8a shows the Langmuir model fitting of experimental data at various temperatures. At different temperatures, the equilibrium adsorption capacities were substantially magnified at low equilibrium concentrations, and then adsorption capacities gradually rose. The rate of Pb^2+^ adsorption is related to the driving force (concentration gradient between Pb^2+^ ions in the solution and adsorbent) and mass transfer coefficient. Following the adsorption process, filling the active site occurs. So mass transfer driving force decreases, and the rate of mass transfer (rate of adsorption) will be decreased. Finally, with filling all of the active sites, the equilibrium reaches^64^. The promising adsorption diagram was supported by the convex climbing deflection of non-linear adsorption isotherms. The adsorption capacity reduced as the temperature rose, which was due to the exothermic nature of adsorption.

The Langmuir, Freundlich, and Simps isotherm models were used to investigate the adsorption mechanism of Pb^2+^ on AHCop, at 25 °C as shown in Fig. 8b. Table 7 shows the parameters of the adsorption isotherm. In terms of the correlation constant (R^2^), the Freundlich model performed poorly when compared to the Langmuir and Sips models.

**Table 7:** Isotherm parameters of Pb^2+^ adsorption on the AHC_op_ (T = 25 °C).

Isotherm	Parameter	Value
$Langmuir {q}_{eq}=\frac{{q}_{max}{K}_{L}{C}_{e}}{1+{K}_{L}{C}_{e}}$	${q}_{max}$ (mg/g)	90.1
	${K}_{L}$ (L/mg)	2.820
	${R}^{2}$	0.998
	R^2^_adj_	0.997
	RSS	189.804
	${\upchi }_{5}^{2}$	2.069
$Freundlich {q}_{eq}={K}_{F}{C}_{e}^\frac{1}{n}$	$K_{F} \frac{{\frac{{{\text{mg}}}}{{\text{g}}}}}{{\left( {\frac{{{\text{mg}}}}{{\text{L}}}} \right)^{n} }}$	61.83
	$n$	11.236
	${R}^{2}$	0.820
	R^2^_adj_	0.730
	RSS	1300.910
	${\upchi }_{5}^{2}$	4.680
$Sips {q}_{eq}=\frac{{q}_{max}{K}_{s}{C}_{e}^\frac{1}{n}}{1+ {K}_{s}{C}_{e}^\frac{1}{n}}$	${q}_{max}$ (mg/g)	86.28
	${K}_{s}$ (L/mg)	2.877
	$\frac{1}{n}$	1.041
	${R}^{2}$	0.971
	R^2^_adj_	0.956
	RSS	372.811
	${\upchi }_{5}^{2}$	1.178

As shown in Table 7, the Langmuir isotherm model had a better correlation coefficient (R^2^ = 0.99) and lower RSS value compared to Sips and Freundlich models. In addition, Langmuir model had higher reduced chi square value rather than Sips model (1.76 times). This higher, χ^2^_red_ value associated with difference between the predicted adsorption capacity (using Langmuir models) and real adsorption capacity at the lower concentration. The Langmuir and Sips models showed acceptable fit with the experimental data. Langmuir and Sips models described better experimental data in the higher and lower concentration respectively. The maximum adsorption capacity (q_max_), calculated by the Langmuir model at 25 °C, was 90.1 (mg/g). The Sips model simplified the Freundlich and Langmuir models at low and high adsorbate concentrations, as seen in Fig. 8b. The heterogeneity coefficient (1/n) in the Sips model was 1.04 in this study, confirming the Langmuir model's better match^65^.

#### Thermodynamic parameters

The thermodynamic characteristics of Pb^2+^ on AHCop at three different temperatures (25, 35, and 45 °C) were calculated using the Van't Hoff equation^66^, and the results are shown in Table 8. The amount of Pb^2+^ adsorption on AHCop decreased with increasing temperature, as seen in Fig. 8b, showing that Pb^2+^ adsorption on AHCop was intrinsically exothermic, as corroborated by estimated value (Table 8). The negative amount of $\Delta {G}^{0}$ showed that the adsorption of Pb^2+^ on AHCop was a spontaneous process. Also, the positive amount of $\Delta {S}^{0}$ implies that the degree of randomness at the solid-solution interface was increased.

**Table 8:** Thermodynamic parameters of Pb^2+^ adsorption onto AHC_op_.

Sample	$\Delta {H}^{0} \left(\frac{{\text{kj}}}{{\text{mol}}}\right)$	$\Delta {S}^{0} \left(\frac{{\text{j}}}{{\text{mol}} \, {\text{K}}}\right)$	$\Delta {G}^{0} \left(\frac{{\text{kj}}}{{\text{mol}}}\right)$			${R}^{2}$
			298 K	308 K	318 K	
AHCop	− 17.6	+ 2.68	− 2.568	− 2.738	− 2.920	0.9691

## Conclusions

The optimum process conditions (temperature of 180 °C, water-biomass ratio of 5 (w/w), ZnCl_2_-biomass ratio of 3.5 (w/w), and resident time of 11.5 h) for Pb^2+^ adsorption on the AHCs were discovered. In optimum conditions, the equilibrium adsorption capacity of 92.24 ± 3.41 (mg/g) was discovered. The Langmuir and Sips models finely fit adsorption isotherms, according to isotherm investigations. Pb^2+^ adsorption kinetics were altered to fit the PSO model, and thermodynamic tests indicated that Pb^2+^ adsorption on AHCop was a simultaneously exothermic process. FTIR study revealed the production of oxygenated functional groups after HTC and KOH activation.

The important process in pb^2+^ adsorption, were electrostatic interactions and complex formation. The structural alterations caused by the HTC process were cleared by SEM and XRD examination of the samples.

## Materials and methods

### Materials and chemicals

Sugarcane bagasse was provided by the Karun sugar industry located in Shoshtar City, Ahvaz state, Iran. Bagasse (BG) was gently rinsed with water many times before being dried in a 105 °C oven for 24 h. Thereafter, the BG was crushed and sieved to a particle size of less than 35 mesh.

All the compounds and substances utilized in the current experiment were of analytical grade. Appropriate amount of Pb(NO_3_)_2_ was dissolved in water and then diluted to appropriate concentrations to make a stock pb^2+^ solution (2000 mg/L).

### Synthesis of bagasse based hydrochar adsorbent

Hydrothermal carbonization (HTC) studies were carried out in a stainless steel autoclave reactor using 200 mL teflon containers. First, 75 mL of distilled water was introduced to the reactor, along with various amounts of biomass and ZnCl_2_. The reactor was then exposed to various times and temperatures with a heating rate of 10 °C/min to determine the impact of the hydrothermal conditions on the hydrochar properties. The reactor was cooled at ambient temperature, and the solid product was separated and rinsed multiple times with distilled water, then dried at 105 °C for 24 h and labeled as HC.

The HC was chemically modified by mixing 2 g of HC with 200 mL of 2 M KOH solution for 1 h at ambient temperature (25 ± 0.5 °C). Next, the modified hydrochar (AHC) was separated, washed with deionized water, and neutralized with a 0.1 M HNO_3_/KOH solution. Afterward, AHC was dried in an oven at 105 °C for 24 h.

### Experimental design

Temperature, resident time, water to biomass, and catalyst to biomass ratio all affect the yield and adsorption capability of HC generated by HTC. So temperature, resident time, water to biomass, and catalyst to biomass ratio are selected as independent variables. Modified adsorption capacity is used as the response value because the Pb^2+^ adsorption capacity per amount of BG is affected by HC's adsorption capacity and yield. Using traditional experimental methods, several experiments were required to investigate these factors. As a result, response surface methodology (RSM) was used in conjunction with central composite design (CCD) to optimize the HTC process. 5 levels of 30 experiments were used with Design-expert (Version 8.0.6). The independent variables were temperature (× 1), resident time (× 2), water to biomass ratio (× 3), and ZnCl_2_ to biomass ratio (× 4) and the response was modified adsorption capacity (MAC). The variable ranges are determined regarding other research. Furthermore, the lower water to biomass ratio limit is used for technical feasibility (complete immersion of biomass in the water environment). Table 9 shows the variable ranges. MAC is calculated by Eq. (2):

**Table 9:** Variable range in the central composite design.

Independent variable	Unit	Coded value				
		− 2	− 1	0	+ 1	+ 2
		Real value				
Temperature	°C	155	180	105	230	255
Resident time	h	1	4.5	8	11.5	15
Water to biomass ratio	g/g	2.5	5	7.5	10	12.5
ZnCl_2_ to biomass ratio	g/g	0.5	1.5	2.5	3.5	4.5

2 $$MAC={Q}_{e}*{Y}_{HC}$$

where ${Q}_{e}$ is the equilibrium adsorption capacity of AHCs and ${Y}_{HC}$ is the yield of HCs at different conditions.

Design-expert (Version 8.0.6) was employed to randomize the tests, which were divided into five stages and 30 experiments. The responses of the variables were adjusted to the quadratic regression model using the equation:

3 $${Y}_{i}={A}_{0}+\sum_{i=1}^{4}{A}_{i}{x}_{i}+\sum_{i=1}^{4}{A}_{ii}{x}_{i}^{2}+ \sum_{1\le i\le j}^{4}{A}_{ij}{x}_{i}{x}_{j}+{\varepsilon }_{i}$$

In Eq. (3), ${Y}_{i}$ is predicted response, ${x}_{i}$ and ${x}_{j}$ are independent variables. ${A}_{0}$, ${A}_{i}$, ${A}_{ii}$, and ${A}_{ij}$ are the average response, linear, quadratic and interaction coefficient effect, respectively and ${\varepsilon }_{i}$ is a random error^67^. The analysis of variance (ANOVA) was employed for model and independent variable significance investigation.

### Characterization of bagasse and hydrochars

The amount of ash in the product was calculated using American Society for Testing and Materials (ASTM E1755-01) guidelines. The chemical composition (C, H, N, S) was determined using an elemental analyzer (Vario EL III, Elementar). A Micromeritics Quantachrome equipment was used to determine the BET surface area at 77 K. To assess the effect of both carbonization and activation processes on surface chemistry, FTIR spectra were obtained using a Cary 630 FT-IR spectrometer in the wavenumber range of 4000 cm^−1^ to 400 cm^−1^. A Hitachi S-2700 scanning electron microscope (SEM) was used to examine the morphology of the samples (SEM). To investigate the properties of thermal decomposition, a TG-DTG (METTLER TOLEDO, TGA2) analysis of HC was performed. The HC and BG were burned with ambient air from room temperature to 800 °C (10 °C/min, 50 mL/min). On an X-ray diffractometer (D/MAX2200, Rigaku, Japan), samples' X-ray diffraction (XRD) patterns were observed using Ni-filtered Cu K radiation (= 1.54). The samples' XRD values were measured in the 5° to 80° range. To determine the point of zero charge (pH_pzc_), 0.1 g of adsorbent was added to 50 mL of KNO_3_ solution and agitated for 24 h at 250 rpm. In the range of 2 to 10, the initial pH value of the solution was adjusted using KNO_3_/KOH (0.01 molar). Then the suspension was filtered, the final pH was measured, and the difference between initial and final pH values versus initial pH values was plotted.

### Batch adsorption of Pb^2+^

For finding the optimum adsorbent, the AHC dose of 1 g/L was added to 250 mL Erlenmeyer flask comprising 100 mL of standard Pb^2+^ solution (200 mg/L) (initial pH value of 6 ± 0.1). All samples were put on the shaker and shaken at ambient temperature (25 ± 0.1 °C) for 24 h. The effect of pH on the adsorption was examined in the pH varies from 2.0 to 7.0. Experimental batch kinetics were conducted (initial pH value of 6 ± 0.1) at various contact times (5 to 240 min), contacting 1 g/L of optimum AHC (AHCop) and 100 mL of Pb^2+^ solution (200 mg/L). Pb^2+^ solutions were diluted with distilled water from the stock solution. For adsorption isotherm studies, 100 mL of lead solutions with initial concentrations vary from (20–200 mg/L) and initial pH value of (6 ± 0.1) was shaken with 1 g/L AHCop for 6 h (200 rpm) at different temperatures (25, 35, and 45 °C). The concentration of Pb^2+^ solutions was tested with an inductively coupled plasma-optical emission spectrometry (ICP-OES 730-ES, Varian, USA). All Pb^2+^ solutions were serially diluted from stock solution (200 mg/L).

The amounts of Pb^2+^ adsorbed in mg/g at various time ${q}_{t}$ were determined by Eq. (4)

4 $${q}_{t}=\frac{{C}_{0}-{C}_{t}}{m}*V$$

where $V$ is the solution volume (mL), $m$ is the quantity of adsorbent used (mg), and ${C}_{0}$ and ${C}_{t}$ are the primary and equilibrium concentrations after a time of t ($\frac{mg}{L}$), respectively. At equilibrium conditions, equilibrium adsorptions capacity ${q}_{eq}$ were calculated by the Eq. (4).

Elovich model^68^, Pseudo-first-order model^69^ (PFO), and pseudo-second-order model^70^ (PSO) were applied for kinetics studies as follows:

Elovich model as equation:

5 $${q}_{t}=\frac{1}{b}\mathrm{ln}(a+abt)$$

PFO model as equation:

6 $${q}_{t}={q}_{eq}[1-\mathrm{exp}\left(1-{k}_{1}t\right)]$$

PSO model as equation:

7 $$\frac{\mathrm{t}}{{q}_{t}}=\frac{1}{{k}_{2}{q}_{eq}^{2}}+\frac{1}{q}_{eq}t$$

where t is time (min), k_1_ (1/min) and k_2_ ($\frac{{\text{g}}}{{\text{mg}} {{\text{min}}}^{-1}}$) are rate coefficients of PFO and PSO, respectively. b ($\frac{{\text{g}}}{{\text{mg}}}$) and a ($\frac{{\text{mg}}}{{\text{g}} {\text{min}}}$) are constant of Elovich model.

Langmuir^71^, Freundlich^72^, and Sips^73^ models were applied for isotherm studies as follows:

Langmuir model:

8 $$\frac{{C}_{e}}{{q}_{eq}}=\frac{1}{{q}_{max}{K}_{L}}+\frac{{C}_{e}}{{q}_{max}}$$

Freundlich model:

9 $$\mathrm{ln}{q}_{eq}=ln{K}_{F}+\frac{1}{{n}_{f}}\mathrm{ln}{C}_{e}$$

Sips model:

10 $${q}_{eq}=\frac{{q}_{max}{K}_{s}{C}_{e}^{\frac{1}{{n}_{s}}}}{1+ {K}_{s}{C}_{e}^{\frac{1}{{n}_{s}}}}$$

where ${q}_{max}$ (mg/g) is maximum adsorption capacity, 1/n_s_ is intensity of adsorption sites,${K}_{L}$ (mL/mg) is equilibrium constant and ${K}_{F}$ (mL/mg) is the coefficient of adsorption capacity.

## Supplementary Information

Supplementary Information.
