# Supplementary material for: Optimized production, Pb(II) adsorption and characterization of alkali modified hydrochar from sugarcane bagasse
Source: Sci Rep. 2021 Nov 16;11:22328. doi: 10.1038/s41598-021-01825-y (PMC8595365; doi:10.1038/s41598-021-01825-y)
Supplement: Supplementary file 1 — Supplementary Information. [file 41598_2021_1825_MOESM1_ESM.docx]

**Appendix A. Supplementary data**

**Table S1. Analysis of variance for variables: temperature (A), water to biomass ratio (B), ZnCl_2_ to biomass ratio (C) and resident time (D).**

| variables | Sum of squares | df | Mean squares | F value | P value |
| --- | --- | --- | --- | --- | --- |
| A(temperature) | *1329.86* | *1* | 1329.86 | 83.80 | < 0.0001 |
| B(water to biomass ratio) | *394.71* | *1* | 394.71 | 24.87 | *0.0002* |
| C(ZnCl_2_ to biomass ratio) | *333.24* | *1* | 333.24 | 21.00 | *0.0004* |
| D(time) | *738.71* | *1* | 738.71 | 46.55 | *< 0.0001* |
| AB | *55.61* | *1* | 55.61 | 3.50 | *0.0822* |
| AC | *27.33* | *1* | 27.33 | 1.72 | *0.2105* |
| AD | *343.64* | *1* | 343.64 | 21.65 | *0.0004* |
| BC | *10.26* | *1* | 10.26 | 0.65 | *0.4349* |
| BD | *16.26* | *1* | 16.26 | 1.02 | *0.3286* |
| CD | *27.43* | *1* | 27.43 | 1.73 | *0.2097* |
| A^2^ | *2564.07* | *1* | 2564.07 | 161.57 | *< 0.0001* |
| B^2^ | *28.33* | *1* | 28.33 | 1.79 | *0.2028* |
| C^2^ | *89.50* | *1* | 89.50 | 5.64 | *0.0324* |
| D^2^ | *915.58* | *1* | 915.58 | 57.70 | *< 0.0001* |
| Residual | 222.17 | 14 | 15.87 |  |  |

**Table S2. Analysis of variance for response model.**

| Source | Sum of squares | Degree of freedom | Mean square | F value | P | Remark | |
| --- | --- | --- | --- | --- | --- | --- | --- |
| Model | 6008.08 | 14 | 429.15 | 27.04 | < 0.0001 | significant | |
| Residues | 222.17 | 14 | 15.87 |  |  |  | |
| Lack of fit | 158.71 | *9* | 17.63 | 1.39 | 0.37 | Not significant | |
| Pure error | 63.46 | *5* | 12.69 |  |  |  | |
| Total | 6230.26 | 28 |  |  |  |  | |
| R^2^ | 0.9643 |  |  |  |  |  | |
| Adj R^2^ | 0.9287 |  |  |  |  |  | |
| Pred R^2^ | 0.8205 |  |  |  |  |  |  |

| 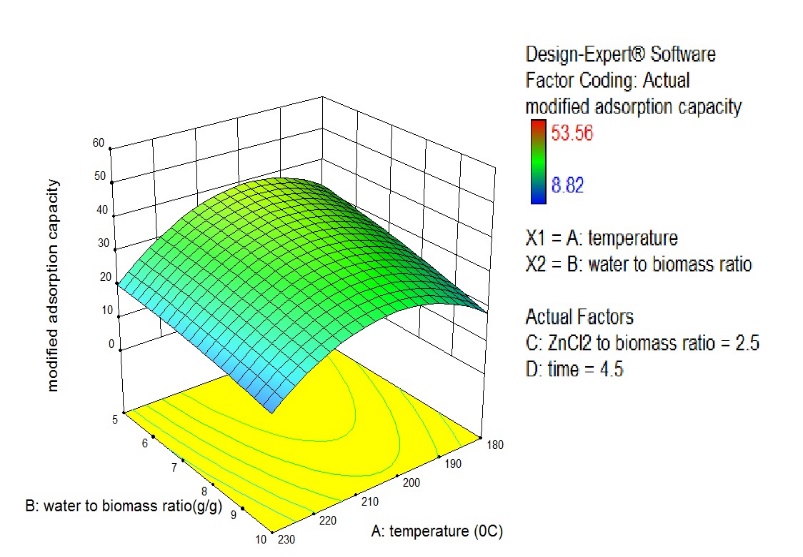 | 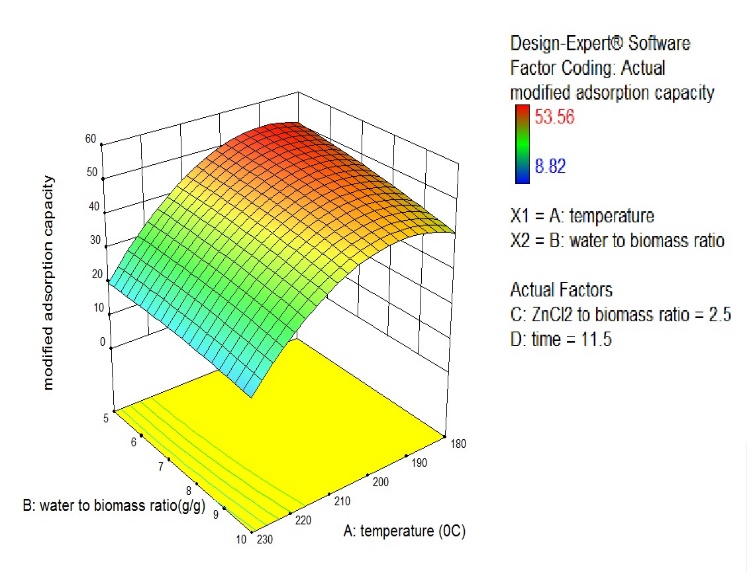 |
| --- | --- |
| 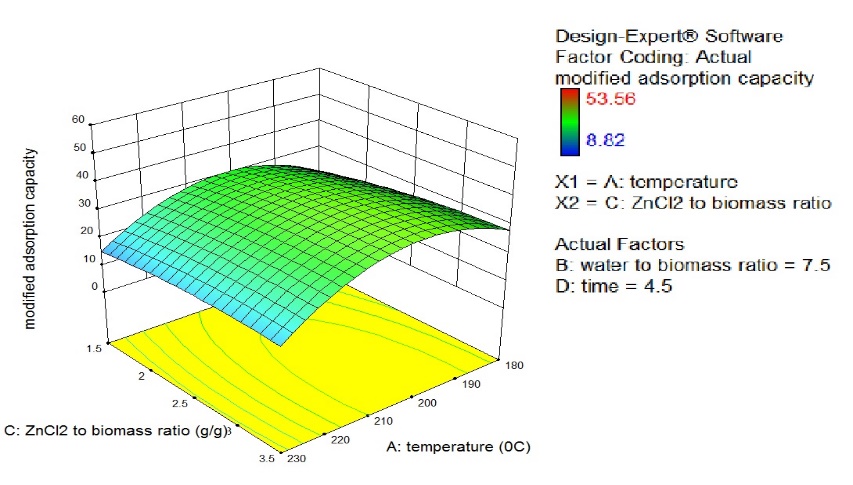 | 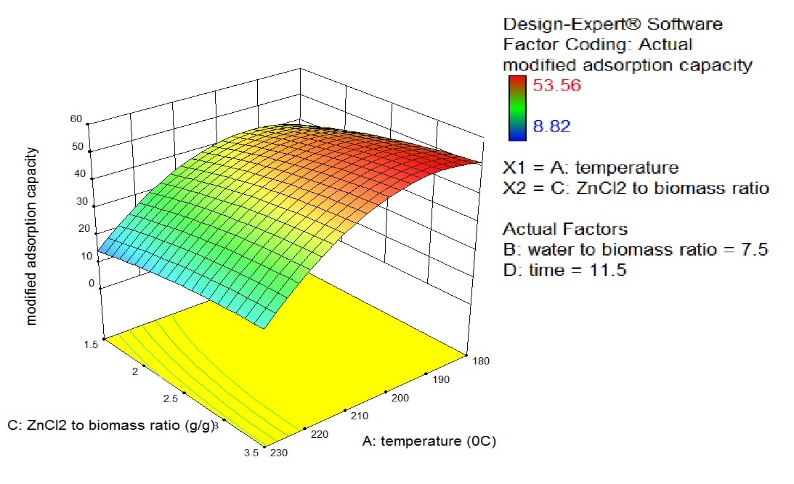 |
| 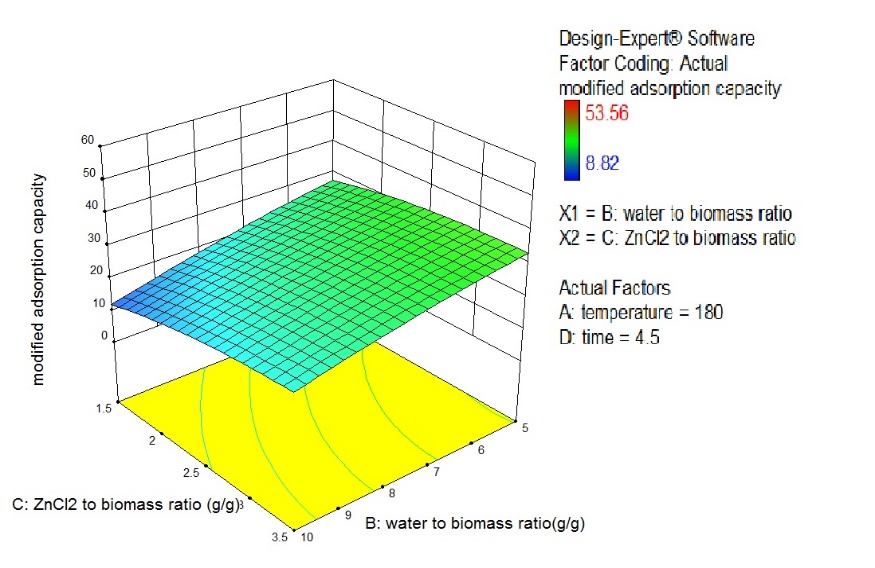 | 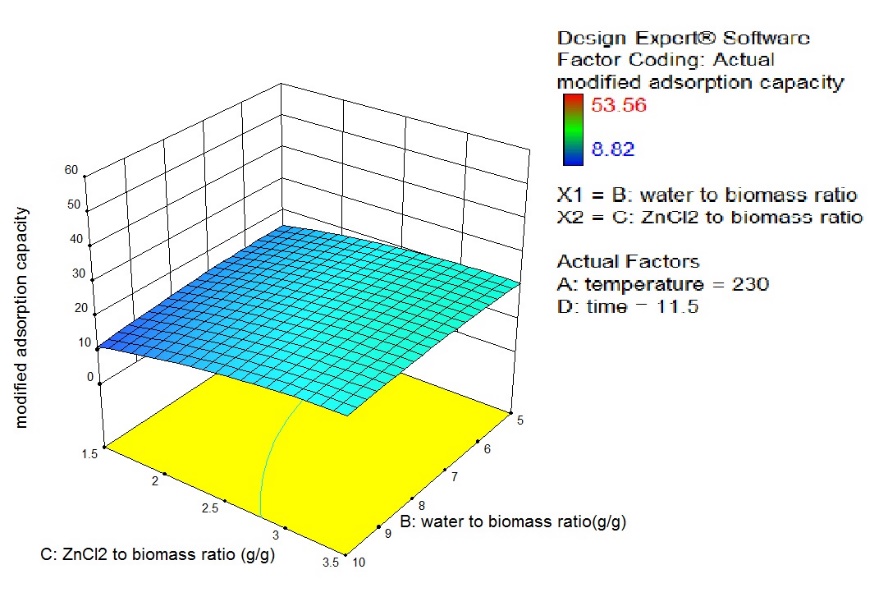 |
| 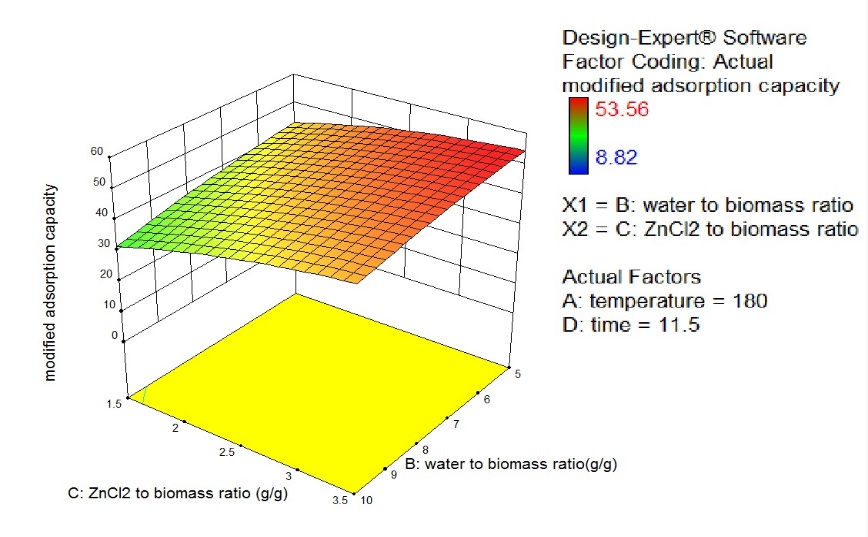 | 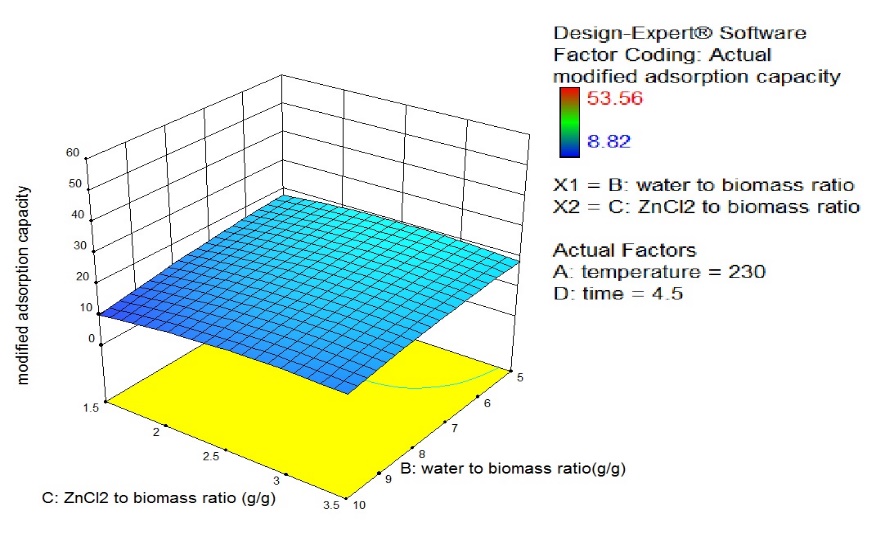 |
| 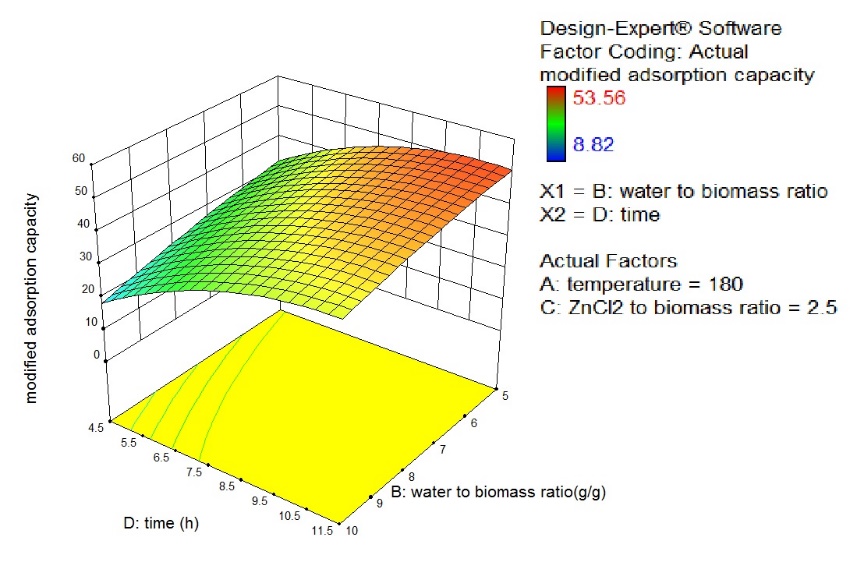 | 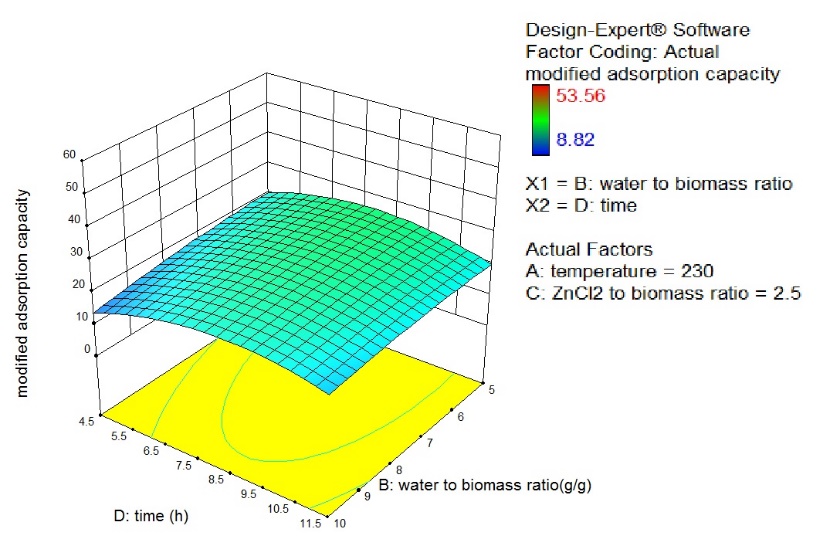 |
| 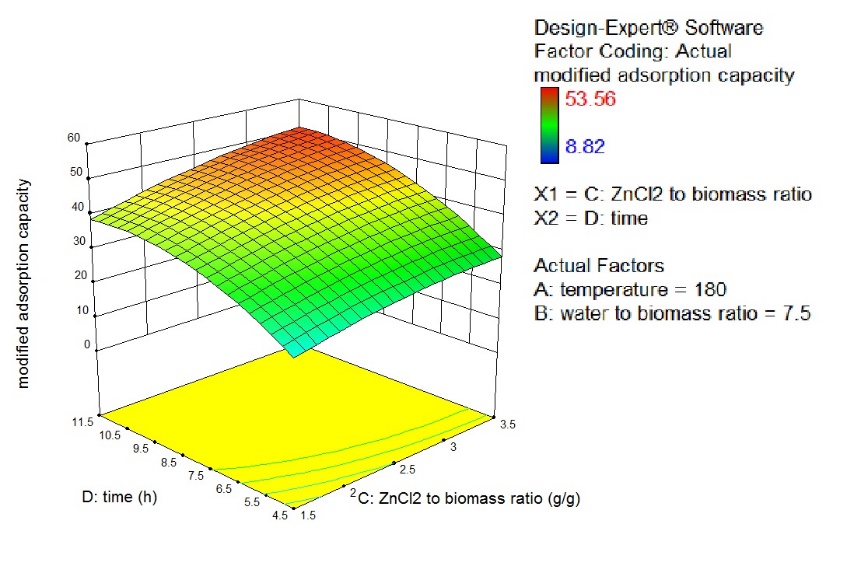 | 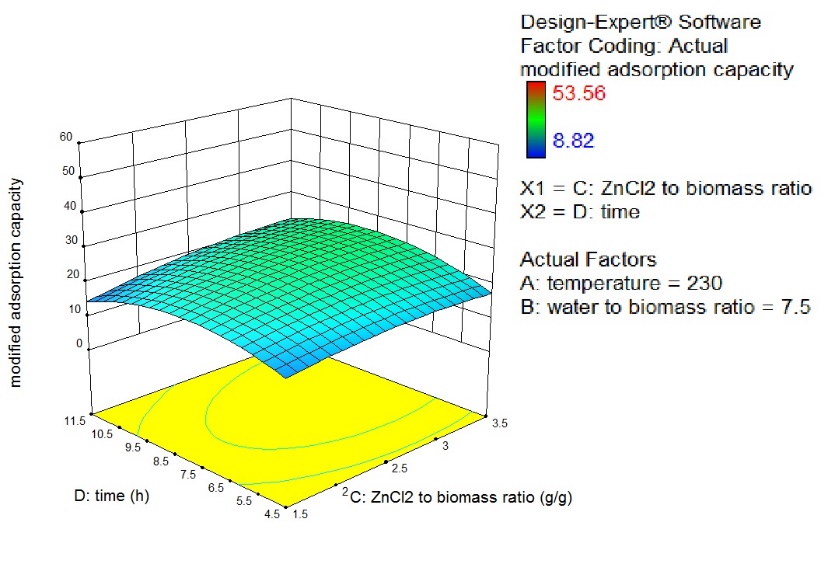 |
| Fig. S1: Three-dimensional response surface for MAC | |

| 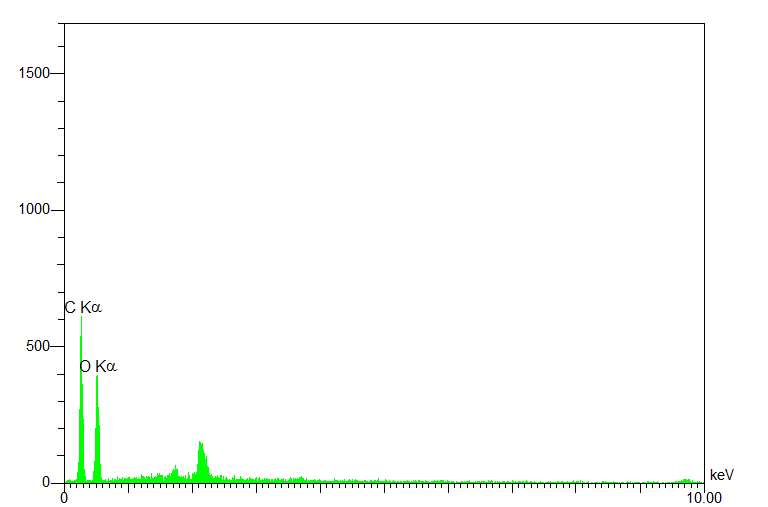  (a) | 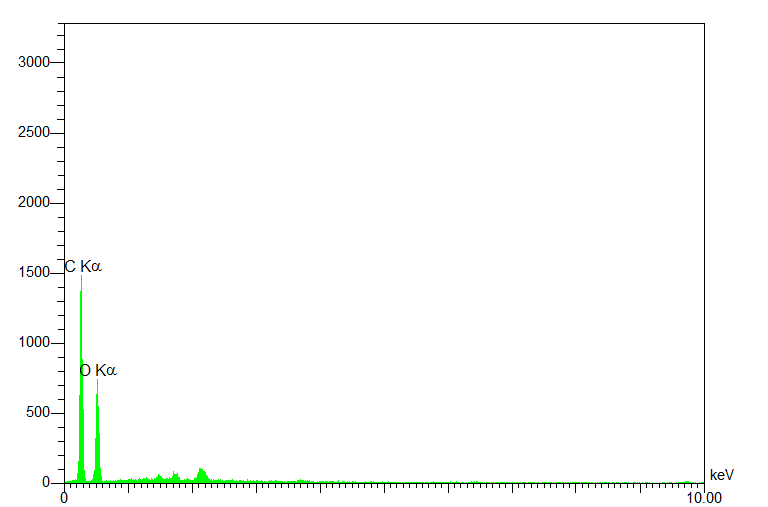  (b) |
| --- | --- |
| 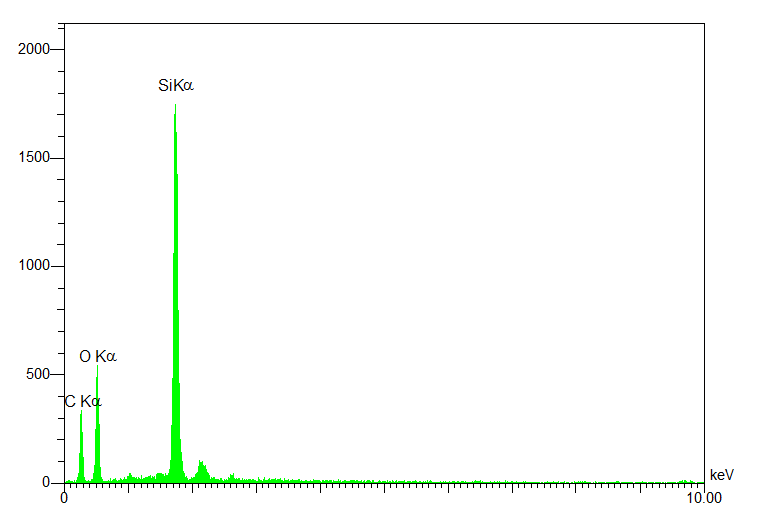  (c) | 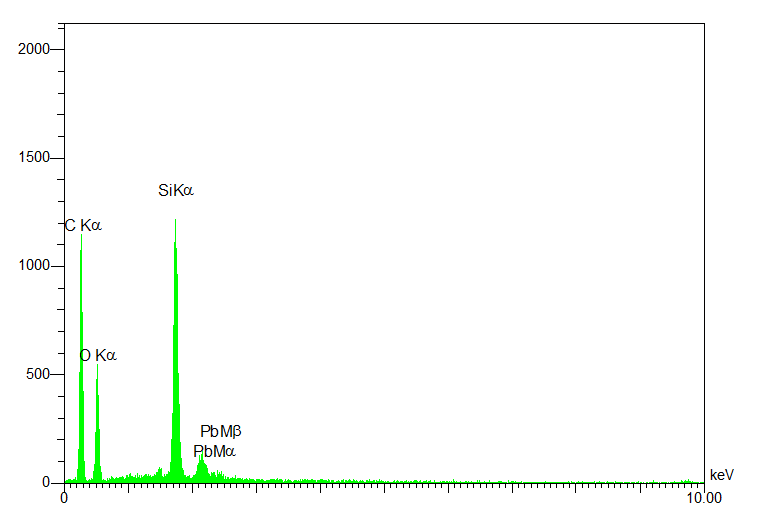  (d) |
| 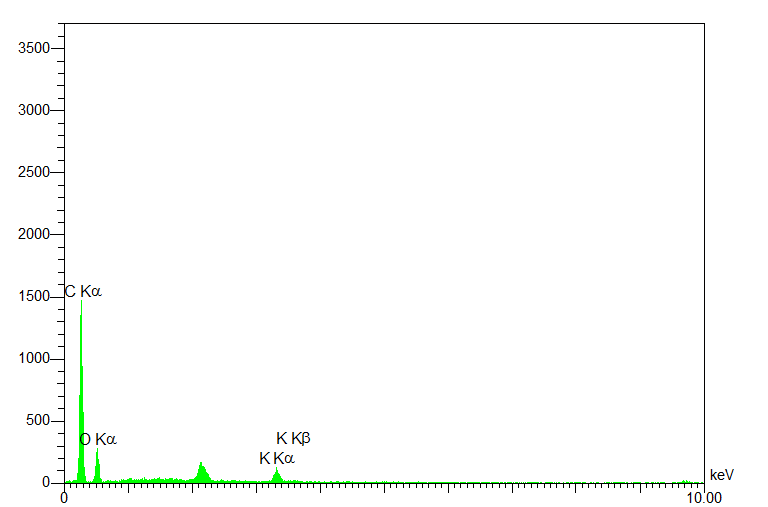  (e) | 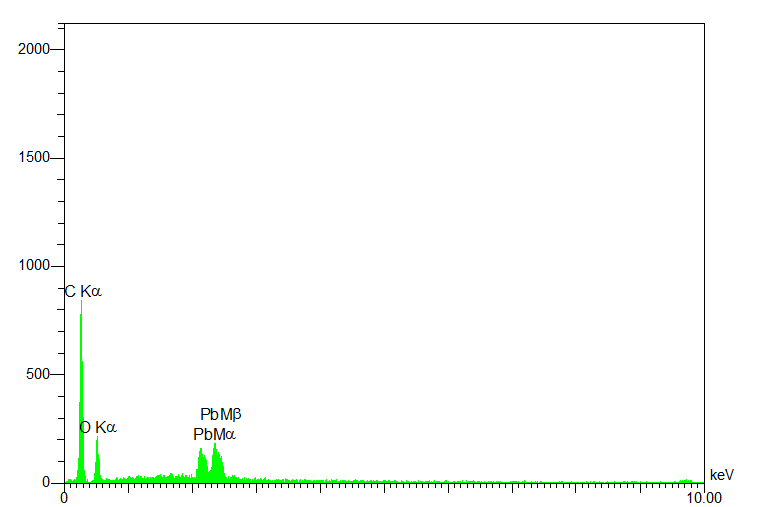  (f) |
| Fig. S2: EDX spectra of (a) bagasse, (b) activated bagasse, (c) HCop , (d) HCop after Pb(II) adsorption, (e) AHCop and (f) AHCop after adsorption. | |

| 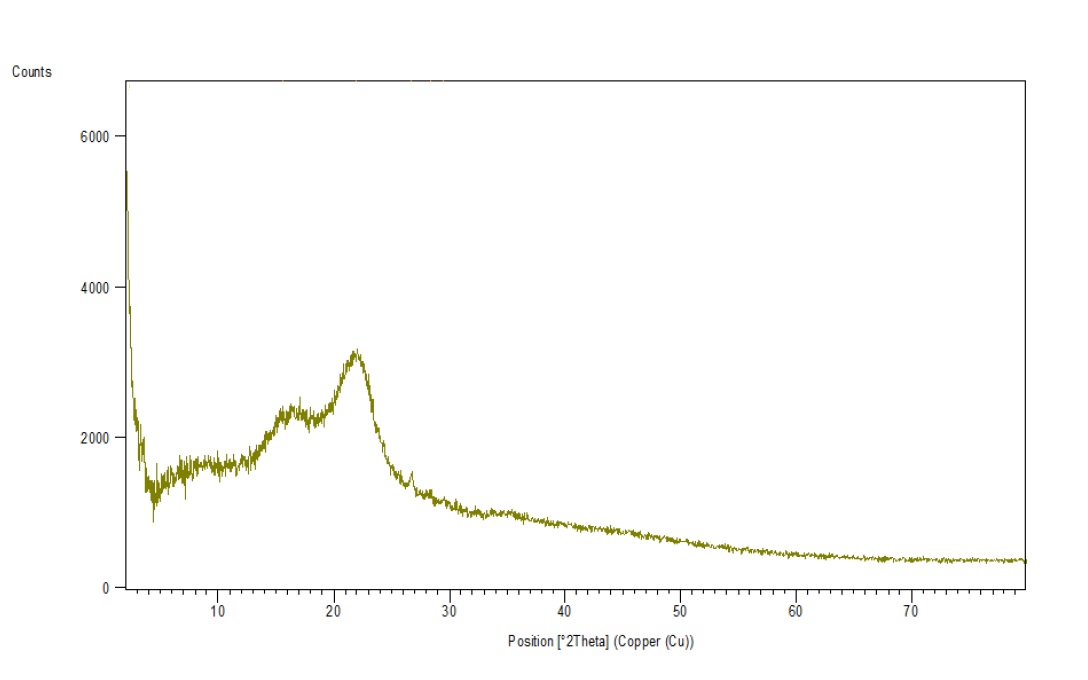  (a) |
| --- |
| 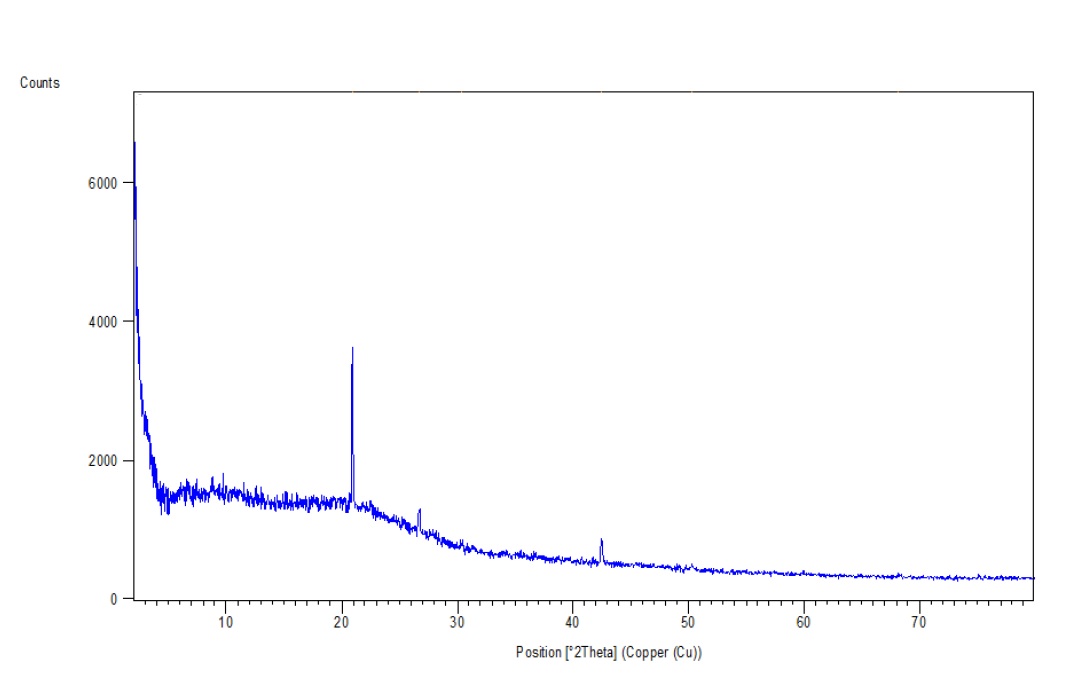  (b) |
| 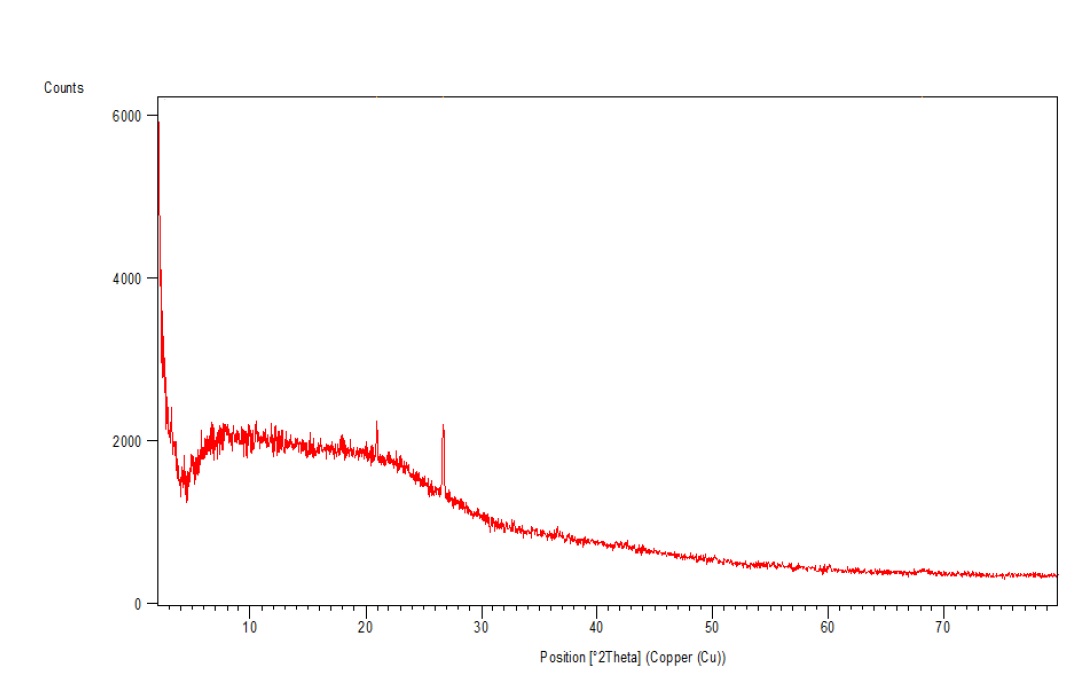  (c)  Fig. S3 XRD diagram of (a) BG, (b) HC_op_ and (c) AHC_op_ |
